# Platelet proteomic signatures of amyloid β-positive mild cognitive impairment and Alzheimer’s disease

**DOI:** 10.1186/s13041-026-01294-2

**Published:** 2026-03-28

**Authors:** Yeong Eun Cho, Andrew Kim, Hyeong Min Lee, Jae Won Oh, Sang Joon Son, Hyun Woong Roh, Yi-Sook Jung, Chang Hyung Hong, Sang Yoon Lee, Kwang Pyo Kim

**Affiliations:** 1 Department of Applied Chemistry, Institute of Natural Science, Kyung Hee University, 1732 Deogyeong-daero, Giheung-Gu, Yongin-Si, Gyeonggi-Do 17104 Republic of Korea; 2 Department of Molecular Medicine & Biopharmaceutical Science, Graduate School of Convergence Science and Technology, Seoul National University, Seoul, 03080 Republic of Korea; 3 Department of Psychiatry, Ajou University School of Medicine, 206 Worldcup-Ro, Yeongtong-Gu, Suwon-Si, Gyeonggi-Do 6499 Republic of Korea; 4 College of Pharmacy, Research Institute of Pharmaceutical Sciences and Technology, Ajou University, Suwon, 16499 Republic of Korea; 5 Department of Biomedical Sciences, Ajou University Graduate School of Medicine, 206 Worldcup-Ro, Yeongtong-Gu, Suwon-Si, Gyeonggi-Do 16499 Republic of Korea; 6 Institute of Medical Science, Ajou University School of Medicine, Suwon, 16499 Republic of Korea; 7 Department of Biomedical Science and Technology, Kyung Hee Medical Science Research Institute, Kyung Hee University, Seoul, 02447 Republic of Korea

**Keywords:** Alzheimer’s disease, Amyloid β, Mild cognitive impairment, Platelets, Proteomics

## Abstract

Early detection of Alzheimer’s disease (AD) is critical for preventing disease progression. Blood platelets have emerged as a useful peripheral source for AD diagnosis. However, the identification of proteomics-based platelet biomarkers of mild cognitive impairment (MCI) and AD in relation to amyloid β (Aβ) deposition remains largely unexplored. In this study, we compared four groups from 18 participants: subjective memory impairment (SMI, *n* = 4) as cognitive normal controls, MCI without Aβ deposition (MCI-A(+), *n* = 5), MCI with Aβ deposition (MCI-A(−), *n* = 5), and AD (*n* = 4). We conducted in-depth platelet protein profiling using high-throughput LC–MS/MS with tandem mass tag labeling. Among the total 4,524 proteins detected, we identified both unique and overlapping differentially expressed proteins in MCI-A(+), MCI-A(−), and AD compared with SMI. Hierarchical clustering analysis revealed seven distinct patterns of proteomic alterations across groups. Functional network and gene ontology enrichment analyses indicated that each cluster was associated with specific processes, including platelet activation, AD, and apoptotic signaling pathways. Notably, upregulated proteins in MCI-A(−) and AD were linked to endomembrane system organization. Furthermore, we quantified the relative abundance of multiple protein candidates that were significantly altered in MCI-A(−) and AD compared with SMI and MCI-A(+). Our findings highlight several platelet proteins–ATP6V0C, AP4B1, RAB2B, PSMD9, FKBP1B, and mTOR–as potential molecular targets for predicting AD at the stage of MCI with Aβ deposition, providing new insights into amyloid-related neurodegeneration.

**Supplementary Information:**

The online version contains supplementary material available at 10.1186/s13041-026-01294-2.

## Introduction

Alzheimer’s disease (AD) is a fatal neurodegenerative disorder characterized by progressive memory loss and cognitive dysfunction. Pathologically, it is marked by senile plaques composed of amyloid β (Aβ) peptide aggregates and neurofibrillary tangles formed by hyperphosphorylated tau protein in the brain [7, 47, 66]. AD is the most common form of dementia and its prevalence continues to rise among elderly populations worldwide. However, no effective therapeutic cure is currently available [18, 57]. AD pathologies progress slowly from a pre-symptomatic stage to a symptomatic stage over a prolonged period [31]. Notably, Aβ and tau proteinopathies, along with abnormal structural brain changes, can occur prior to the clinical onset of AD [31].

The pathological changes of AD are generally irreversible, making it difficult to treat once the disease has progressed to its later stages [37]. Therefore, earlier detection at the pre-symptomatic or prodromal stages is considered an efficient strategy for preventing or delaying AD onset [1]. Mild cognitive impairment (MCI) is regarded as a prodromal stage between normal cognition and dementia, and its symptoms are recognized as an important risk factor for AD [9, 43]. Numerous studies have sought to develop effective measures to predict the conversion of MCI to AD, relying on well-established AD biomarkers such as Aβ, tau, and apolipoprotein genotype [30, 32]. Given that Aβ deposition is a hallmark of AD, individuals with MCI who harbor an Aβ burden are considered more vulnerable to AD progression with a higher probability.

Positron emission tomography (PET) imaging of brain Aβ deposits, magnetic resonance imaging, and biochemical measurements of Aβ as well as phosphorylated and total tau in cerebrospinal fluid have been widely utilized for accurate and reliable diagnosis of AD [4, 26, 42, 66]. However, brain imaging requires expensive equipment and incurs high costs, while cerebrospinal fluid collection is highly invasive, imposing considerable limitations on their routine clinical use. Liquid chromatography–tandem mass spectrometry (LC–MS/MS) has emerged as a powerful proteomic methodology that enables large-scale analysis of protein expression changes [45]. High resolution proteomics provides sensitive detection and quantitative evaluation of broad and complex proteome alterations. Numerous proteomic studies have identified diagnostic and prognostic biomarkers of AD using more readily accessible samples, such as patient-derived blood plasma and platelets [19, 51, 71].

A growing body of evidence has demonstrated the functional involvements of anucleated blood platelets in AD [14, 61, 65]. Platelets mediate neurotransmitter secretion and uptake similar to neurons and serve as a major peripheral source of circulating amyloid precursor protein (APP) [10, 44, 50]. APP in platelets is cleaved by β-secretase and γ-secretase, generating Aβ through the same amyloidogenic pathway as in the brain [49, 50, 73]. Activated platelets secret significant amounts of Aβ into the blood, which in turn can further promote platelet activation [13, 14, 36]. Notably, platelet-derived Aβ can cross into the brain under conditions of blood–brain barrier dysfunction, leading to Aβ deposition in the cerebral cortex. Thus, platelet activation is increasingly recognized as a critical risk factor contributing to AD pathologies [13, 14, 36, 68]. Nevertheless, platelet protein biomarkers associated with Aβ positivity in MCI and AD remain largely unknown.

In this study, we explored proteomic alterations in patient platelet samples from four groups: subjective memory impairment (SMI) as a normal control, MCI without Aβ deposition (MCI-A(+)), MCI with Aβ deposition (MCI-A(−)), and AD, all classified by Aβ-PET imaging. Differentially expressed proteins (DEPs) in MCI-A(+), MCI-A(−), and AD groups relative to the SMI group were analyzed using LC–MS/MS with tandem mass tag (TMT) labeling. Bioinformatic analyses were then performed to characterized the functional roles, biological pathways, and distinctive expression patterns of the DEPs, leading to identification of potential platelet biomarkers that may predict AD during the MCI stage with Aβ deposition.

## Materials and methods

### Study participants

All participants underwent diagnostic evaluation with cognitive function tests, including the clinical dementia rating (CDR), sum of boxes of CDR (CDR-SB), global deterioration scale (GDS), and mini-mental state examination (MMSE). In addition, the Seoul neuropsychological screening battery (SNSB) was administered to assess attention, language, visuospatial function, memory, and frontal/executive function. All cognitive assessments and Aβ-PET imaging were conducted in the Department of Psychiatry, Ajou University Hospital, in accordance with protocols approved by the Institutional Review Board (AJRB-BMR-KSP-18–038).

### Aβ-PET imaging

To evaluate Aβ deposition, Aβ-PET imaging was performed to obtain quantitative measures of Aβ pathology, following previously described procedures [56]. In brief, all participants received an intravenous bolus injection of ^18^F-flutemetamol (mean dose: 185 MBq) into the antecubital vein. A 20-min PET scan was acquired 90 min after injection. The ^18^F-flutemetamol PET scans were co-registered with each participant’s computed tomography (CT) scan. Cortical amyloid burden was quantified using a Discovery STe PET/CT scanner (GE Healthcare, Milwaukee, WI, USA) under identical settings. Aβ positivity was determined by a nuclear medicine physician with more than 10 years of experience, based on visual rating.

### Platelet isolation and protein extraction

Peripheral blood samples were collected from all participants and platelets were isolated according to previously described procedures [34]. Platelet-rich plasma (PRP) was obtained by centrifugation at 100 × *g* for 10 min at room temperature without brake. The upper PRP fraction was carefully transferred to a new tube and centrifuged at 1,000 × *g* for 10 min to pellet platelets. The platelet pellet was washed once with phosphate-buffered saline (PBS) and immediately subjected to protein extraction. No platelet activation inhibitors were added during the isolation procedure. All samples were processed using an identical protocol with minimal handling time and standardized centrifugation conditions to minimize ex vivo platelet activation and ensure comparability across diagnostic groups. Platelets were lysed in RIPA buffer (150 mM NaCl, 50 mM triethylammonium bicarbonate, 1% SDS, 0.5% dichloroacetic acid, pH 8) for 20 min on ice to extract total proteins. Lysates were clarified by centrifugation at 15,000 × *g* for 20 min at 4 °C, and the resulting supernatants were collected and used as the final platelet lysates. Protein concentrations were determined using the Pierce™ BCA Protein Assay Kit (Thermo Fisher Scientific, Waltham, MA, USA).

### Trypsin digestion and peptide preparation

Protein extracts (200 µg) were dissolved in 300 µl of buffer containing 4% SDS, 100 mM dithiothreitol, and 100 mM Tris–HCl (pH 7.6). Samples were loaded onto Microcon-30 kDa centrifugal filter units with Ultracel-30 membranes (Merck, Rahway, NJ, USA) and centrifuged (14,000 × *g*, 60 min, room temperature). The use of a 30 kDa molecular weight cut-off filter was chosen to prioritize efficient removal of MS-incompatible detergents and reproducible digestion, while maintaining consistency across all samples for relative quantitative analysis. The filters were washed three times with 180 µl of 8 M urea in 0.1 M Tris–HCl (pH 8.5) by centrifugation (14,000 × *g*, 40 min), followed by alkylation with 50 mM iodoacetamide for 30 min at 37 °C. After alkylation, the filters were centrifuged and washed twice with 180 µl of 8 M urea in 0.1 M Tris–HCl (pH 8.5), and three times with 180 µl of 100 mM triethylammonium bicarbonate (TEAB). Trypsin (Thermo Fisher Scientific) dissolved in 50 mM TEAB was added to the filters at an enzyme-to-protein ratio of 1:50 (w/w), and samples were incubated overnight at 37 °C. The resulting tryptic peptides were eluted by centrifugation three times with 75 μl of 100 mM TEAB (14,000 × *g*, 20 min). Eluted peptides were vacuum-dried and desalted using Macro SpinColumns™ (Harvard Apparatus, Holliston, MA, USA) prior to mass spectrometry analysis.

### TMT labeling and fractionation

Peptides were labeled with TMT 10-plex (Thermo Fisher Scientific) according to the manufacturer’s instructions. Briefly, 41 µl of anhydrous acetonitrile (ACN) was added to each tube, vortexed, and centrifuged. Each sample in 0.1 M TEAB was mixed with a distinct TMT reagent in ACN and incubated for 1 h at room temperature. The reaction was quenched with 5% hydroxylamine for 15 min. Two TMT 10-plex sets were used; platelet samples from patients were assigned to nine channels, and the remaining channel contained pooled samples serving as a reference. To increase protein identification in the data-dependent acquisition (DDA) method, samples were separated into 96 fractions using an 1100 series high performance liquid chromatography (HPLC) system (Agilent Technologies, Wilmington, DE, USA) equipped with an XBridge BEH C18 column (130 Å, 5 µm, 4.6 mm × 250 mm; Waters, Milford, MA, USA). Solvent A (4.5 mM ammonium formate in 2% ACN, pH 10–11) and solvent B (4.5 mM ammonium formate in 90% ACN, pH 10–11) were applied with the following gradients: 0–7 min, 0% B; 7–12 min, 12% B; 12–35 min, 25% B; 35–85 min, 40% B; 85–95 min, 70% B; 95–101 min, 70% B; 101–105 min, 0% B; 105–115 min, 0% B. The 96 fractions were subsequently concatenated into 12 fractions. The fractionated peptides were vacuum-dried and desalted using Macro SpinColumns™.

### LC–MS/MS analysis

Desalted peptide samples were dissolved in 0.1% formic acid and analyzed using a nanoACQUITY UPLC System (Waters, Milford, MA, USA) coupled to a Q Exactive Orbitrap mass spectrometer (Thermo Fisher Scientific, Waltham, MA, USA). Samples were separated on a 180-min gradient using solvent A (3% ACN/0.1% formic acid in water) and solvent B (90% ACN/0.1% formic acid). The gradient was as follows: 0–5 min, 2% B; 5–10 min, 10% B; 10–140 min, 25% B; 140–160 min, 40% B; 160–161 min, 90% B; 161–165 min, 90% B; 165–166 min, 2% B; 166–180 min, 2% B. The flow rate was maintained at 0.300 µl/min. Peptides were loaded onto a C18 trap column (75 µm × 2 cm, 3 µm particle size, 100 Å pore size, Thermo Fisher Scientific) and ionized on an EASY-Spray analytic column (75 µm × 500 mm, 2 µm particle size, 100 Å, Thermo Fisher Scientific). Proteome profiling was performed in DDA mode. Full MS spectra were acquired in positive ion mode with a scan rage of 350–1800 m/z, resolution of 70,000, automatic gain control (AGC) target value of 3e6, and maximum ion-injection time of 100 ms. MS/MS scans were acquired at a resolution of 35,000, with an AGC target value of 1e6 and a maximum ion injection time of 120 ms. The dynamic exclusion time was set to 30 s, the high collisional dissociation collision energy was set to 32, and the isolation window was set to 1.6 m/z.

### Proteome data processing

MS/MS spectra were analyzed using the SEQUEST HT search engine in Proteome Discoverer 2.1 software (Thermo Fisher Scientific) against the UniProtKB human protein database (UniProtKB/SwissProt with 20,324 entries). Search parameters specified trypsin digestion with cleavage at arginine and lysine residues, allowing up to two missed cleavages. Carbamidomethylation of cysteine, TMT on lysine, and the N-terminus were set as static modifications, while methionine oxidation was specified as a dynamic modification. Precursor mass tolerance was set to 10 ppm and fragment mass tolerance to 0.02 Da. Peptide–spectrum matches and protein identifications were filtered using a 1% false discovery rate (FDR). Proteins were quantified only when supported by at least two unique peptides. Reporter ion quantification was based on intensity. The two TMT sets were normalized to the reference channel, and each channel was corrected according to the ratio of summed protein abundances across all proteins identified in all channels.

### Bioinformatics and statistical analysis

Proteome data were filtered to include only proteins identified in all TMT channels. Partial least squares–discriminant analysis (PLS–DA) was performed using MetaboAnalyst 5.0. DEPs with *p*-value < 0.1 were analyzed using the Database for Annotation, Visualization and Integrated Discovery (DAVID) and g:Profiler, based on gene ontology (GO), Kyoto Encyclopedia of Genes and Genomes (KEGG), and Reactome databases, to assess related biological functions (Benjamini–Hochberg FDR < 0.05). Hierarchical clustering and pattern analysis were conducted in Perseus ver1.6.1.1 using *z*-score- normalized data with Pearson correlation. Receiver operating characteristic (ROC) curves for multiple biomarker candidates were generated in GraphPad Prism 9.4.1 software (La Jolla, CA, USA) using Wilson/Brown method. Volcano plots, pathway enrichment plots, and box plots were also created with GraphPad Prism 9.4.1. For effect size and statistical power estimation, participants were additionally stratified according to Aβ pathology status into Aβ-positive (MCI-A(−)+ AD, *n* = 9) and Aβ-negative (SMI+ MCI-A(+), *n* = 9) groups. This classification reflects the primary biological variable underlying the study hypothesis, namely Aβ deposition, rather than clinical diagnostic category alone. Standardized mean differences (Cohen’s *d*) were calculated for selected candidate proteins. Post hoc statistical power was estimated under a two-tailed α level of 0.05 based on the observed effect sizes and sample size.

## Results

### Diagnosis on Aβ-negative and -positive MCI and AD

We recruited newly enrolled participants comprising four in the SMI group, five in the MCI-A(+) group, five in the MCI-A(−) group, and four in the AD group. The group classification was based on cognitive function tests and Aβ-PET imaging. Cognitive decline was evaluated using general function tests, including CDR, CDR-SB, GDS, and MMSE (Table 1). Test scores indicated normal condition for SMI, mild decline for MCI, and severe decline for AD. The SNSB results, as illustrated by radar charts of cognitive domain scores for attention, language, visuospatial function, memory, and frontal/executive function represented similar cognitive functional status across groups (Supplementary Fig. S1). In addition, we performed PET imaging in all participants to examine the Aβ deposits in the brain. The Aβ-PET imaging results demonstrated higher levels of Aβ accumulation in MCI-A(−) and AD groups compared with the SMI and MCI-A(+) groups (Supplementary Fig. S1).

**Table 1:** Demographic data of the study participants (*n* = 18)

Characteristic	SMI (*n* = 4)	MCI-A(+) (*n* = 5)	MCI-A(−) (*n* = 5)	AD (*n* = 4)
Age, mean (range)	73.25 (67–78)	75.2 (71–78)	75 (73–79)	72.25 (64–81)
Sex (Male, Female)	1 M, 3F	5F	5F	1 M, 3F
CDR	0.5	0.5	0.5	≥ 1
CDR-SB	1.13	2.10	2.40	6.88
GDS	2	2.4	2.4	5.25
MMSE (SD^*^)	27.25 (1.26)	24.60 (2.30)	21.60 (6.80)	13.25 (2.99)
Aβ deposition^**^	negative	negative	positive	positive

^*^SD: standard deviation; ^**^PET imaging results

### Global proteome profiling of platelets

**Fig. 1:**
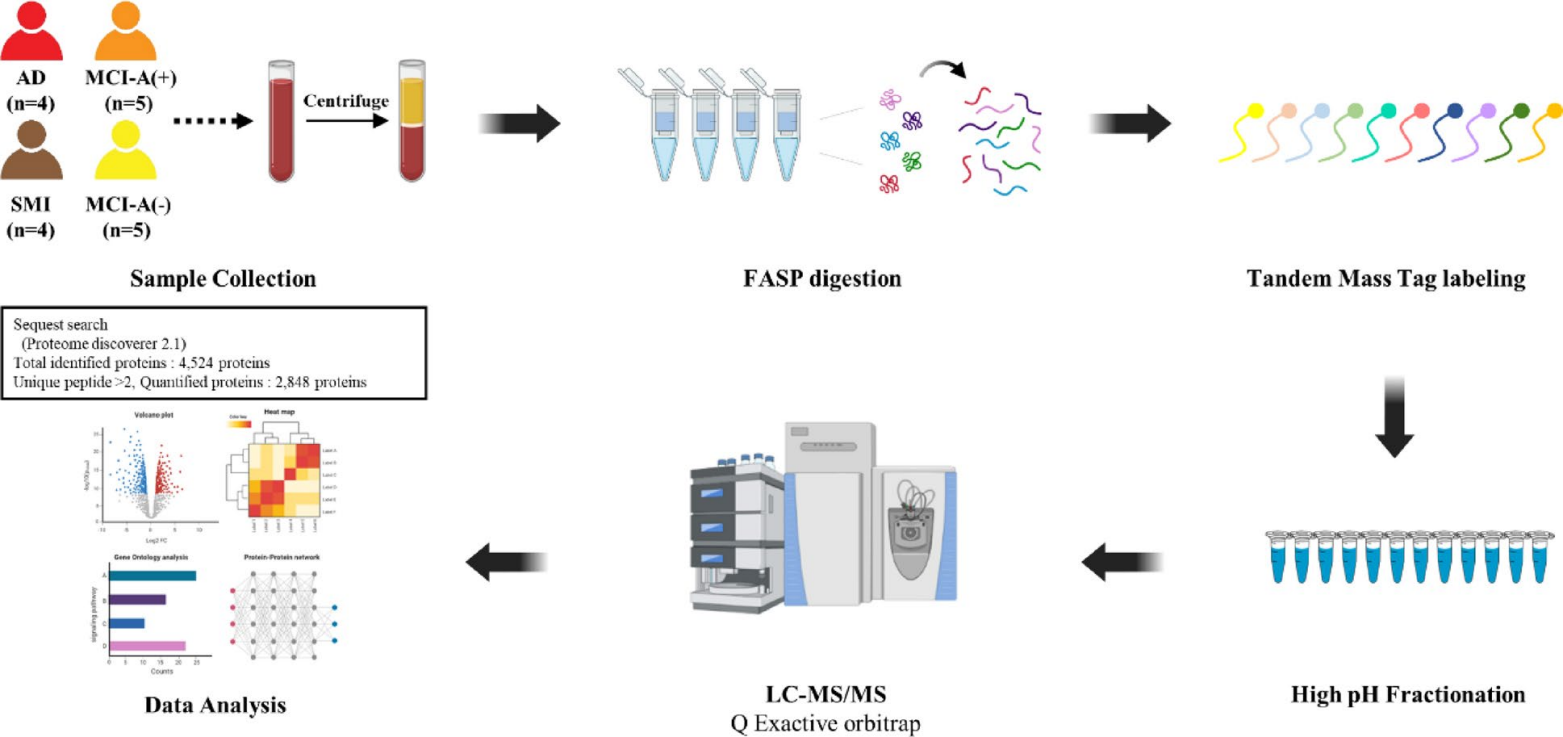
Experimental workflow of platelet proteomics using LC–MS/MS

We performed a comprehensive analysis of platelet samples from all participants using LC–MS/MS, as illustrated in Fig. 1. In total, 4,524 proteins were identified, exceeding those reported in previous platelet proteomic studies [35, 60, 71]. Among these, 2,848 quantifiable proteins were selected for subsequent bioinformatics analyses (Supplementary Table S1). PLS–DA of proteomic profiles from the SMI, MCI-A(+), MCI-A(−), and AD groups demonstrated clear discrimination among groups along the trajectory of disease progression and Aβ deposition (Supplementary Fig. S2). These distinct distributions highlight proteomic signatures that differentiate each disease stage.

### Clustering of proteomic features

**Fig. 2:**
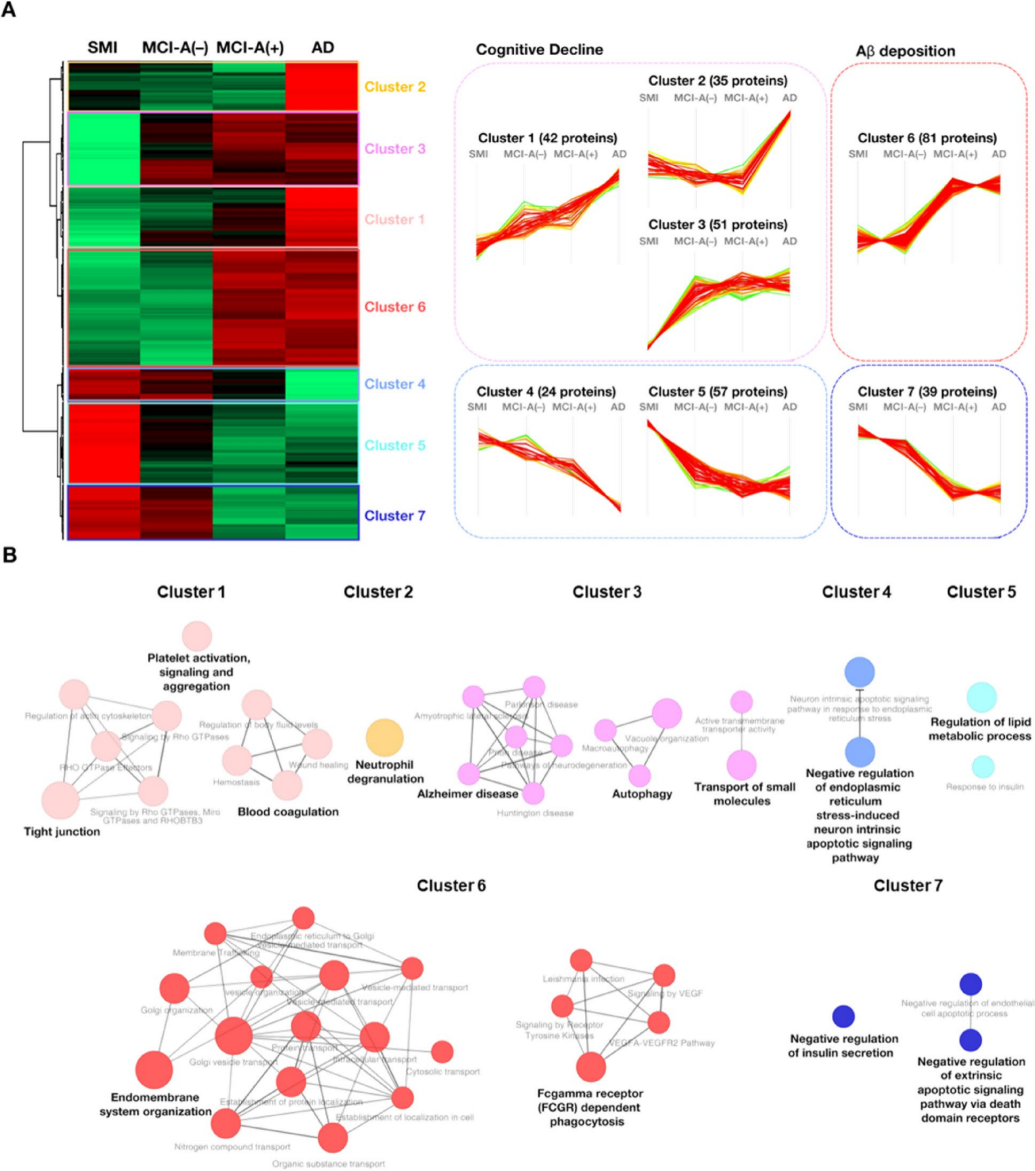
Platelet proteomic patterns and associated biological functions in cognitive decline. **A** Heatmap of hierarchical clustering of platelet proteins across SMI, MCI-A(+), MCI-A(−), and AD, groups into seven clusters. Each cluster represents a distinct expression trend, ranging from proteins increasing with cognitive decline to those associated with Aβ levels. **B** ClueGO network analysis of cluster-associated biological processes. Node sizes reflect statistical significance (*p*-value < 0.05), and networks colors correspond to cluster colors: cluster 1 (pink), cluster 2 (orange), cluster 3 (hot pink), cluster 4 (sky blue), cluster 5 (cyan), cluster 6 (red), and cluster 7 (navy). The networks highlight functional links between proteomic alterations and pathways such as platelet activation, metabolism, and endomembrane system involvement in APP processing

Hierarchical clustering of platelet proteomes was performed to examine global protein expression trends across SMI, MCI, and AD groups. Among 2,848 quantifiable proteins, seven distinct clusters were identified, each representing characteristic expression patterns associated with cognitive decline and Aβ levels (Fig. 2A; Supplementary Table S1). Cluster 1 proteins gradually increased from SMI to AD, whereas cluster 2 proteins were elevated only in AD. Cluster 3 proteins were upregulated in both MCI and AD in accordance with cognitive deterioration. Clusters 4 and 5 displayed a progressive decrease from SMI through MCI to AD, aligning with cognitive impairment. Clusters 6 and 7 showed expression changes directly linked to the presence or absence of Aβ, suggesting potential as markers of Aβ-related alterations.

Biological terms associated with these clusters were mapped using ClueGO in Cytoscape, with significant terms (*p*-value < 0.05) highlighted in Fig. 2B. Clusters 1–3, enriched in proteins upregulated with cognitive decline, were associated with platelet activation and AD processes. These processes are linked to neuroinflammation and vascular changes that exacerbate dementia, with prior studies reporting significant correlations between platelet activity and cognitive assessment scores, including MMSE [53]. Cluster 4, characterized by reduced protein expression in AD, was enriched in terms suggesting suppression of apoptosis-related pathways. Cluster 5 proteins were associated with lipid metabolism and insulin responsiveness, consistent with clinical and experimental evidence that metabolic imbalance worsens neurodegenerative disease symptoms [12]. Disruptions in lipid homeostasis have been implicated in hippocampal injury, altered APP processing, and compromised blood–brain barrier integrity [16]. Moreover, insulin signaling, which is essential for cognitive function, has been linked to AD pathology when dysregulated [5, 15, 17]. Proteins in cluster 6, integral to the endomembrane system, are likely involved in APP processing. APP, a type-1 transmembrane protein that, when misprocessed, leads to the accumulation of Aβ [20, 21]. Reduced expression of cluster 7 proteins in Aβ-positive cases suggests negative regulation of insulin secretion, reminiscent of the metabolic disturbances observed in type 2 diabetes [5, 59].

### Biological functional analysis of proteins associated with cognitive decline

**Fig. 3:**
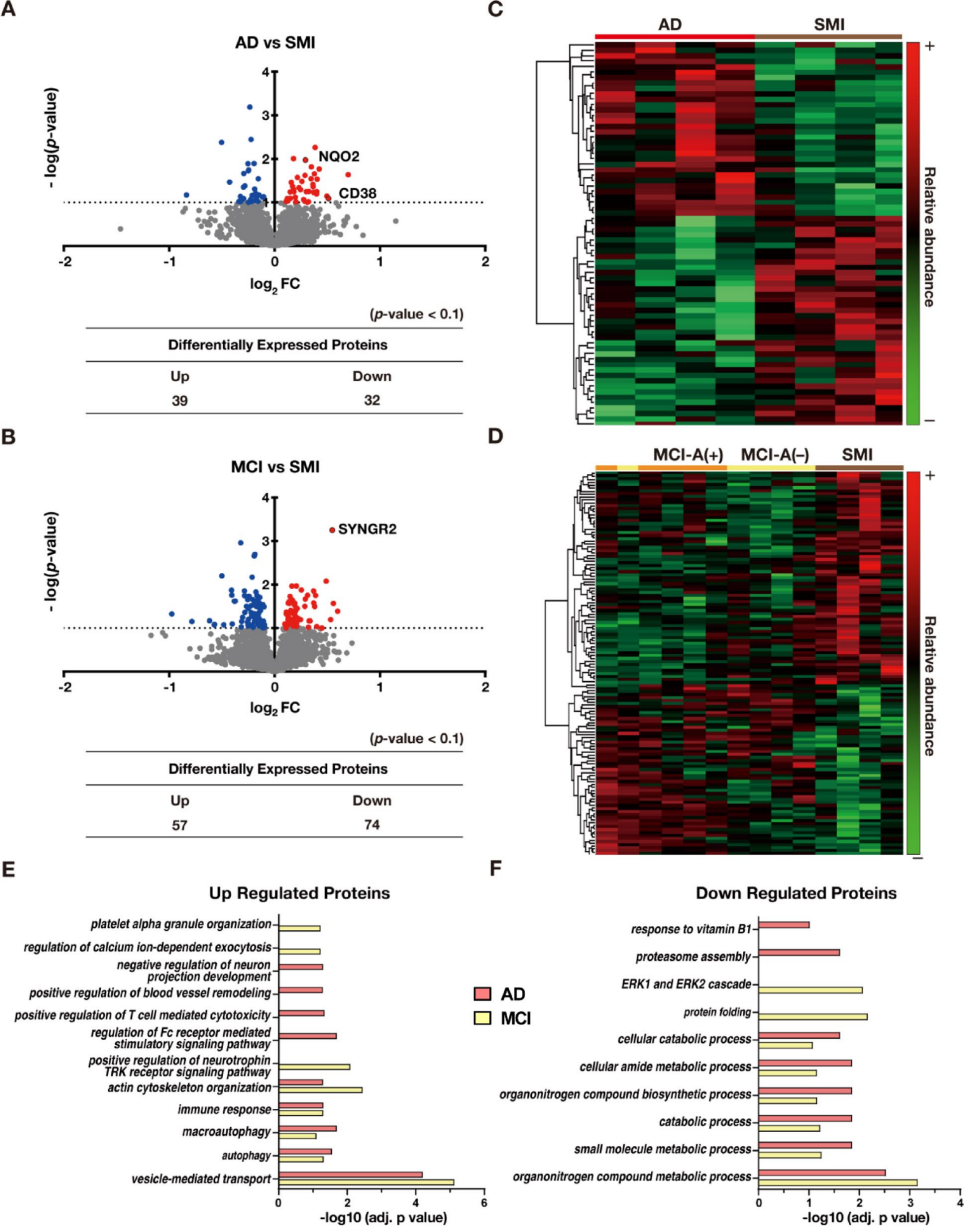
Integrated proteomic profiling and functional analysis in AD and MCI. (A, B) Volcano plots of DEPs. **A** AD versus SMI, highlighting NQO2 and CD38 among significantly altered proteins. **B** Pooled MCI (MCI-A(+) and MCI-A(−)) versus SMI, highlighting SYNGR2 among other DEPs. (C, D) Hierarchical clustering heatmaps. **C** Comparison of AD versus SMI, showing distinct proteomic changes. **D** SMI, MCI-A(+), and MCI-A(−) analyzed together, revealing distinct separation of SMI from the two MCI groups considered jointly, despite the lack of subgroup-specific separation. AD is shown in red, MCI-A(−/+) in orange and yellow, and SMI in brown. (E, F) Bar graphs of GO enrichment analyses. **E** Upregulated proteins in pooled MCI (MCI-A(+) and MCI-A(−)) and AD, enriched in vesicle-mediated transport, autophagy, immune response, and actin cytoskeleton organization. **F** Downregulated proteins in pooled MCI and AD, enriched in metabolic and catabolic processes, with ERK1/2 cascade downregulation specific to MCI

To identify proteins involved in cognitive decline, proteins significantly altered in AD and MCI compared to SMI were analyzed (*p*-value < 0.1). A total of 71 proteins were differentially expressed in the AD group relative to SMI (Fig. 3A; Supplementary Table S2), while 131 proteins were differentially expressed in the MCI group, which included both MCI-A(+) and MCI-A(−) (Fig. 3B; Supplementary Table S2). Among these DEPs, quinone reductase 2 (NQO2), CD38, and synaptogyrin-2 (SYNGR2) are particularly noteworthy, as they have been implicated in the pathogenesis of AD. NQO2, which was notably elevated in AD, is primarily located in the neuronal cytoplasm and functions as a detoxifying cytosolic flavoenzyme. It is thought to play a critical role in memory acquisition and loss [27]. Recent research has revealed that Elevated NQO2 expression has also been associated with multiple psychiatric and neurodegenerative disorders, including schizophrenia, Parkinson’s disease, and AD [55]. CD38 expression increases with aging and contributes to disturbances in energy metabolism and neuroinflammation [25]. In an AD mouse model (APP/PS1), CD38 inhibition improved energy metabolism, reduced proinflammatory cytokine levels, and enhanced learning ability [29]. SYNGR2, a protein that regulates the localization of synaptophysin into synaptic-like microvesicles in neuronal cells [6], was significantly upregulated in both MCI groups, regardless of Aβ deposition.

Hierarchical clustering analysis of DEPs revealed a clear separation between AD and SMI (*p*-value < 0.1; Fig. 3C). However, neither MCI-A(+) nor MCI-A(−) formed a cluster distinct from SMI when analyzed separately. Notably, when SMI, MCI-A(+), and MCI-A(−) were analyzed together, their joint distribution relative to SMI reveals distinct proteomic differences (*p*-value < 0.1, Fig. 3D). This suggests that while the two MCI subgroups are not independently distinguishable from SMI in clustering analyses, their multi-group distribution underscores proteomic alterations associated with early cognitive decline. Furthermore, each MCI subgroup exhibited a unique set of DEPs when compared with SMI (*p*-value < 0.1; Supplementary Fig. S3), highlighting subgroup-specific proteomic changes despite the absence of sharp clustering separation. Importantly, the DEPs shared AD and MCI-A(−) are highlighted as potential biomarkers of Aβ positivity. A comprehensive list of DEPs identified in each MCI group relative to SMI is provided in Supplementary Table S2.

GO enrichment analysis was performed on proteins significantly altered in AD and in the pooled MCI group (MCI-A(+) plus MCI-A(−)) to examine biological functions associated with cognitive impairment. GO biological processes with adjusted *p*-values < 0.1 were identified (Fig. 3E, F). Upregulated proteins in AD and MCI were commonly enriched in vesicle-mediated transport, autophagy, immune response, and actin cytoskeleton organization. Platelet activation is known to promote coagulation, adhesion, cytoskeletal remodeling, and vesicle-mediated transport [62]. Human platelets also express FcγRIIa, a low-affinity Fc receptor for immunoglobulin G that mediates platelet activation [38, 54]. In addition, activation of the tropomyosin receptor kinase (TRK) family of neurotrophin receptors has been shown to induce platelet aggregation [8]. Collectively, these enriched terms implicate platelet function in both MCI and AD. Downregulated proteins in MCI and AD were enriched in metabolic and catabolic processes, with additional enrichment in the extracellular signal-regulated kinase 1/2 (ERK1/2) cascade specifically in MCI. The ERK1/2 cascade plays a central role in adult brain functions such as synaptic plasticity, brain development, and memory formation in the central nervous system, and its dysfunction has been linked to stroke and AD [63].

### Identification of DEPs Correlated with Aβ Positivity

**Fig. 4:**
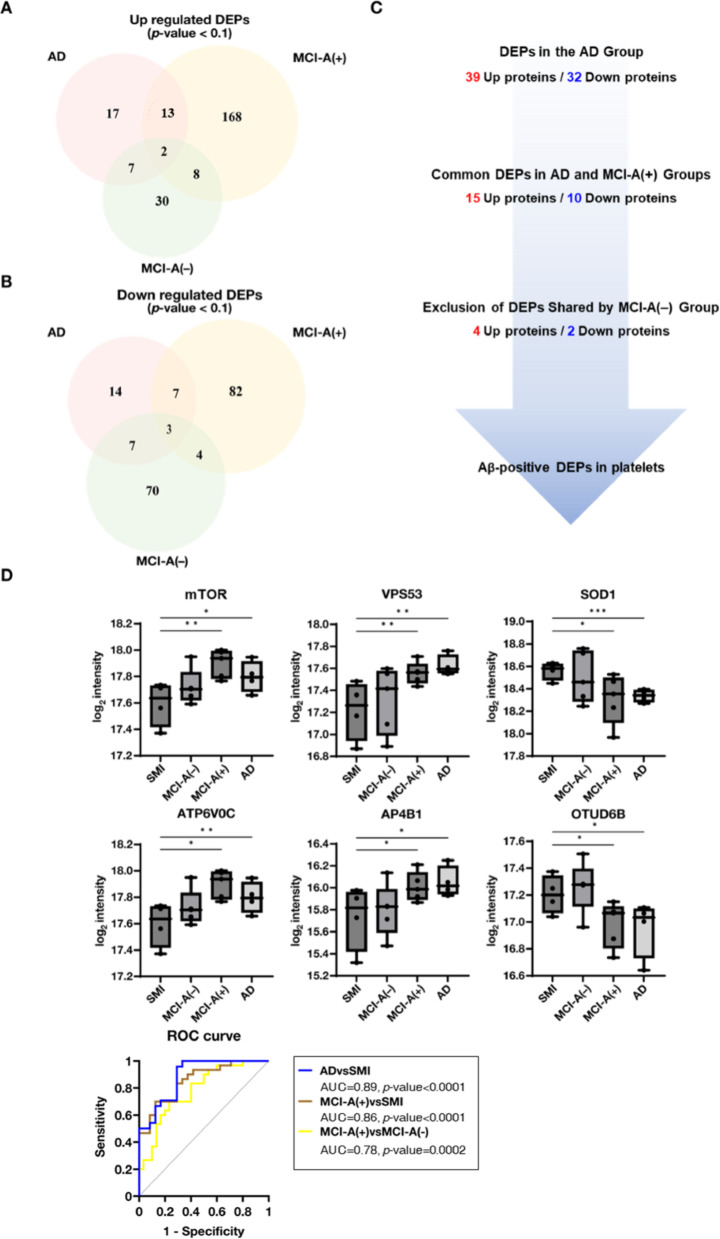
Differential expression and diagnostic analysis of proteins associated with Aβ positivity in AD and MCI. (A, B)Venn diagrams overlapping upregulated **A** and downregulated **B** DEPs among MCI-A(+), MCI-A(−), and AD compared to SMI (*p*-value < 0.1). DEPs shared by AD and MCI-A(−) but absent in MCI-A(+) are circled, indicating their potential association with Aβ deposition. **C** Flow diagram of the DEP filtering strategy, starting with all DEPs in the AD, identifying those shared with MCI-A(−) group, and excluding those shared with MCI-A(+), yielding platelet DEPs specifically associated with Aβ positivity. **D** Box plots of six Aβ-positive proteins (mTOR, VPS53, ATP6V0C, AP4B1, SOD1, OTUD6B) across groups. Significant increases or decreases were observed in MCI-A(−) and AD compared with SMI (^*^p-value < 0.1, ^**^p-value < 0.05, ^***^p-value < 0.01). Lower panel displays ROC curves demonstrating the diagnostic performance of these proteins in distinguishing MCI-A(−) and AD from SMI and MCI-A(+), with AUC values reflecting sensitivity and specificity

To identify DEPs with similar changes in MCI-A(−) and AD but not in MCI-A(+), we compared protein expression across these groups (Supplementary Fig. S3). DEPs associated with Aβ deposition were isolated by excluding those shared with MCI-A(+) (Fig. 4A–C; Table 2). Among the DEPs, proteins showing a significant correlation between Aβ deposition and expression levels were defined as Aβ-positive proteins in platelets. This analysis identified specific proteins as potential indicators of Aβ positivity, including four upregulated proteins: mammalian target of rapamycin (mTOR), vacuolar protein sorting-associated protein 53 homolog (VPS53), V-type proton ATPase 16 kDa proteolipid subunit c (ATP6V0C), and AP-4 complex subunit beta-1 (AP4B1); and two downregulated proteins: Cu/Zn-dependent superoxide dismutase 1 (SOD1) and ovarian tumor domain-containing protein 6B (OTUD6B) (Fig. 4D). These proteins exhibited a significant correlation with Aβ positivity; for example, mTOR was markedly upregulated in both MCI-A(−) and AD. VPS53 is involved in the retrograde transport pathway, mediating endosome-to-Golgi trafficking–a mechanism crucial for APP processing by γ-secretase [33]. In addition, SOD1 interacts with Aβ, disrupting its enzymatic activity and thereby exacerbating oxidative stress and promoting neuronal apoptosis [70]. ROC curve analysis using the combined normalized levels of these six proteins effectively distinguished the AD and MCI-A(−) groups from the MCI-A(+) and SMI groups (Fig. 4D), underscoring their distinct expression patterns associated with Aβ accumulation. To further quantify the magnitude of these group differences in relation to the primary pathological variable, we performed effect size and post hoc power analyses comparing Aβ-positive (MCI-A(−) plus AD, *n* = 9) and Aβ-negative (SMI plus MCI-A(+), *n* = 9) participants. This Aβ-based stratification was applied solely for estimation of effect magnitude and statistical power, while the original four-group comparisons remain unchanged. The selected candidate proteins exhibited large standardized mean differences (Cohen’s *d* = 1.24–1.70), and the corresponding effect size and power estimates are summarized in Supplementary Table S3.

**Table 2:** Biomarkers associated with Aβ-positive cognitive decline, distinguishing AD and MCI-A(−) from MCI-A(+)

Up	AD/SMI		MCI-A(−)/SMI		MCI-A(+)/SMI	
Protein	${Log}_{2}$ FC^*^	*p*-value	${Log}_{2}$ FC	*p*-value	${Log}_{2}$ FC	*p*-value
ATP6V0C	0.42	0.017	0.35	0.090	0.05	0.685
VPS53	0.40	0.029	0.34	0.035	0.09	0.659
STXBP3	0.38	0.022	0.22	0.013	0.16	0.191
CISD2	0.35	0.028	0.16	0.100	0.12	0.507
AP4B1	0.32	0.096	0.28	0.100	0.06	0.728
DNAJB2	0.26	0.033	0.37	0.012	-0.04	0.772
mTOR	0.20	0.097	0.30	0.013	0.13	0.250
PSMD1	0.20	0.076	0.24	0.015	0.02	0.829
VIPAS39	0.18	0.010	0.18	0.004	0.11	0.167
LYPLA2	0.17	0.043	0.25	0.019	0.08	0.343
ARHGEF2	0.16	0.034	0.25	0.066	0.05	0.564
TTC37	0.15	0.081	0.23	0.022	0.05	0.553
PPP2R5D	0.11	0.094	0.12	0.025	0.01	0.911

Down	AD/SMI		MCI-A(−)/SMI		MCI-A(+)/SMI	
Protein	${Log}_{2}$ FC	*p*-value	${Log}_{2}$ FC	*p*-value	${Log}_{2}$ FC	*p*-value
FGG	−0.32	0.096	−0.25	0.026	−0.16	0.132
FAH	−0.29	0.022	−0.19	0.019	−0.14	0.124
OTUD6B	−0.25	0.099	−0.22	0.075	−0.06	0.645
PPCS	−0.23	0.085	−0.22	0.026	−0.14	0.150
SOD1	−0.22	0.004	−0.25	0.070	−0.06	0.650
NECAP2	−0.18	0.061	−0.17	0.047	−0.10	0.166
NUDC	−0.15	0.067	−0.20	0.010	−0.07	0.226

^*^FC: fold change

## Discussion

In this study, we analyzed platelet proteomic data using mass spectrometry, a key tool for protein quantification and biomarker discovery. Our dataset revealed a greater number of identified proteins compared to previous platelet studies in AD and other diseases [35, 60, 71]. Notably, proteins showing significant changes in the cognitive decline groups compared with the SMI group were particularly associated with platelet activation. Previous studies have demonstrated that AD patients with rapid cognitive decline exhibit higher platelet activation levels than those with slower decline during follow-up [61]. Moreover, activated platelets can induce perivascular inflammation, secrete Aβ into the blood, and promote amyloid deposition [13]. We also found a strong association between upregulated DEPs in cognitive decline groups and the neurodegeneration pathway described in KEGG. Among these DEPs, mTOR exhibited significant alterations even in the presence of Aβ, underscoring its crucial role in regulating neuron survival, differentiation, and development via the mTOR signaling pathway [11, 28]. Hyperactivation of this pathway has been observed in postmortem brain tissue of AD patients, and Aβ accumulation is known to further enhance this activation [28, 69].

The seven protein clusters demonstrated distinct expression patterns associated with cognitive impairment and Aβ deposition. We further examined the biological functions of clusters 6 and 7, particularly in relation to the APP amyloidogenic endomembrane system, where APP is cleaved by β-secretase and γ-secretase. In this system, APP undergoes endocytosis and is subsequently processed by β- and γ-secretase, while platelet activation stores Aβ in α-granules and releases it upon stimulation [72]. This pathway, integral to platelet activation and Aβ storage and release, also involves the insulin-degrading enzyme, which competitively regulates Aβ degradation. Within cluster 6, proteins such as Ras-related protein Rab-2B (RAB2B), AP4B1, and ATP6V0C are linked to membrane trafficking, intracellular transport vesicle formation, and organelle acidification–processes essential in AD and other neurodegenerative diseases [2, 3, 40, 46, 64]. ATP6V0C, a component of vacuolar ATPase, maintains the acidification of endosomes and lysosomes [41]. Notably, Aβ oligomer formation is highly pH dependent and accelerates under endo-lysosomal acidic conditions [58]. V-ATPase has therefore emerged as an attractive in age-related neurodegenerative disease, and ATP6V0C specifically plays a dual role in both V-ATPase function and neurotransmitter release [22, 39]. Cluster 7 includes FK506-binding protein 1B (FKBP1B), a member of the peptidyl-prolyl isomerase family, which shows reduced expression in the hippocampus of aging rats and in early AD, potentially impairing insulin secretion [23, 48]. Another member, 26S proteasome non-ATPase regulatory subunit 9 (PSMD9), part of the 26S proteasome, has been associated with insulin deficiency and type 2 diabetes when overexpressed in the pancreas [24, 67]. In summary, this study highlights six proteins within pathways indicative of early-stage AD in Aβ-positive patients. These proteins, potentially integral to Aβ accumulation, warrant further investigation to elucidate their roles in AD progression.

Our study, while identifying platelet protein signatures associated with early AD-related pathological changes, has several limitations that should be acknowledged. The sample size in each diagnostic group was relatively small, reflecting the practical challenges associated with recruiting well-characterized participants undergoing Aβ-PET imaging, a costly and resource-intensive procedure that is particularly difficult to apply in early or prodromal stages such as Aβ-positive MCI. Despite this limitation, all participants were stringently classified based on both Aβ pathology and detailed cognitive assessments, providing a high level of diagnostic certainty and allowing the identification of consistent proteomic patterns across a well-defined disease continuum rather than heterogeneous clinical classifications. To further evaluate the robustness of the observed differences despite the modest sample size, we conducted additional effect size analysis based on Aβ pathology status, the primary biological determinant of interest in this study. Large standardized mean differences were observed between Aβ-positive and Aβ-negative groups (Cohen’s *d* ≈ 1.2–1.7; Supplementary Table S3), supporting the strength of the separation within this dataset. Importantly, this Aβ-based grouping was used exclusively for effect magnitude estimation and does not replace the original four-group comparative analyses. Nevertheless, independent validation in larger, preferably longitudinal, cohorts will be essential to confirm the generalizability and clinical relevance of these findings. Although the cross-sectional design precludes direct assessment of longitudinal disease progression, the identification of platelet proteins showing consistent alterations specifically in Aβ-positive MCI and AD suggests that these biomarkers may reflect early pathological changes associated with disease progression rather than merely descriptive markers of established dementia. Accordingly, the results should be interpreted as exploratory and indicative of candidate platelet biomarkers. Future prospective longitudinal studies following Aβ-defined MCI individuals will be essential to determine conversion rates to AD and to evaluate whether these platelet protein signatures can serve as true prognostic biomarkers rather than cross-sectional correlates of established pathology.

In conclusion, this study advances the understanding of AD by applying platelet proteomic analyses across different stages of dementia, in combination with Aβ-PET imaging and cognitive function assessments. We identified platelet proteins potentially contributing to Aβ accumulation within the endomembrane system. These findings present candidate biomarkers in platelets that may aid in predicting AD during the MCI stage characterized by Aβ positivity.

## Supplementary Information

Below is the link to the electronic supplementary material.

Supplementary Material 1
Aβ-PET images and SNSB results of study participants

Supplementary Material 2
PLS–DA of proteomic profiles from SMI, MCI-A(−), MCI-A(+), and AD groups

Supplementary Material 3
Identification of DEPs in MCI subgroups compared with SMI

Supplementary Material 4
Quantified proteins and peptides in platelets

Supplementary Material 5
DEPs associated with cognitive decline and Aβ among SMI, MCI-A(−), MCI-A(+), and AD groups

Supplementary Material 6
Effect size (Cohen’s d) and post hoc statistical power analysis for selected Aβ-associated candidate proteins comparing Aβ-positive (MCI-A(+) + AD) and Aβ-negative (SMI + MCI-A(−)) participants

## Data Availability

The mass spectrometry proteomics data have been deposited to the ProteomeXchange Consortium via the PRIDE [52] partner repository with the dataset identifier PXD067460.

## Abbreviations

Aβ: Amyloid β
ACN: Acetonitrile
AD: Alzheimer’s disease
AP4B1: AP-4 complex subunit beta-1
APP: Amyloid precursor protein
ATP6V0C: V-type proton ATPase 16 kDa proteolipid subunit c
CDR: Clinical dementia rating
CDR-SB: Sum of boxes of CDR
DEP: Differentially expressed protein
FDR: False discovery rate
FKBP1B: FK506-binding protein 1B
GDS: Global deterioration scale
GO: Gene ontology
KEGG: Kyoto encyclopedia of genes and genomes
LC–MS/MS: Liquid chromatography–tandem mass spectrometry
MCI: Mild cognitive impairment
MCI-A(+): MCI without Aβ deposition
MCI-A(−): MCI with Aβ deposition
MMSE: Mini-mental state examination
mTOR: Mammalian target of rapamycin
OTUD6B: Ovarian tumor domain-containing protein 6B
PET: Positron emission tomography
PSMD9: 26S proteasome non-ATPase regulatory subunit 9
RAB2B: Ras-related protein Rab-2B
ROC: Receiver operating characteristic
SMI: Subjective memory impairment
SNSB: Seoul neuropsychological screening battery
SOD1: Superoxide dismutase 1
TMT: Tandem mass tag
VPS53: Vacuolar protein sorting-associated protein 53 homolog
