# Supplementary figures and images for: Platelet proteomic signatures of amyloid β-positive mild cognitive impairment and Alzheimer’s disease
Source: Mol Brain. 2026 Mar 28;19:33. doi: 10.1186/s13041-026-01294-2 (PMC13151324; doi:10.1186/s13041-026-01294-2)

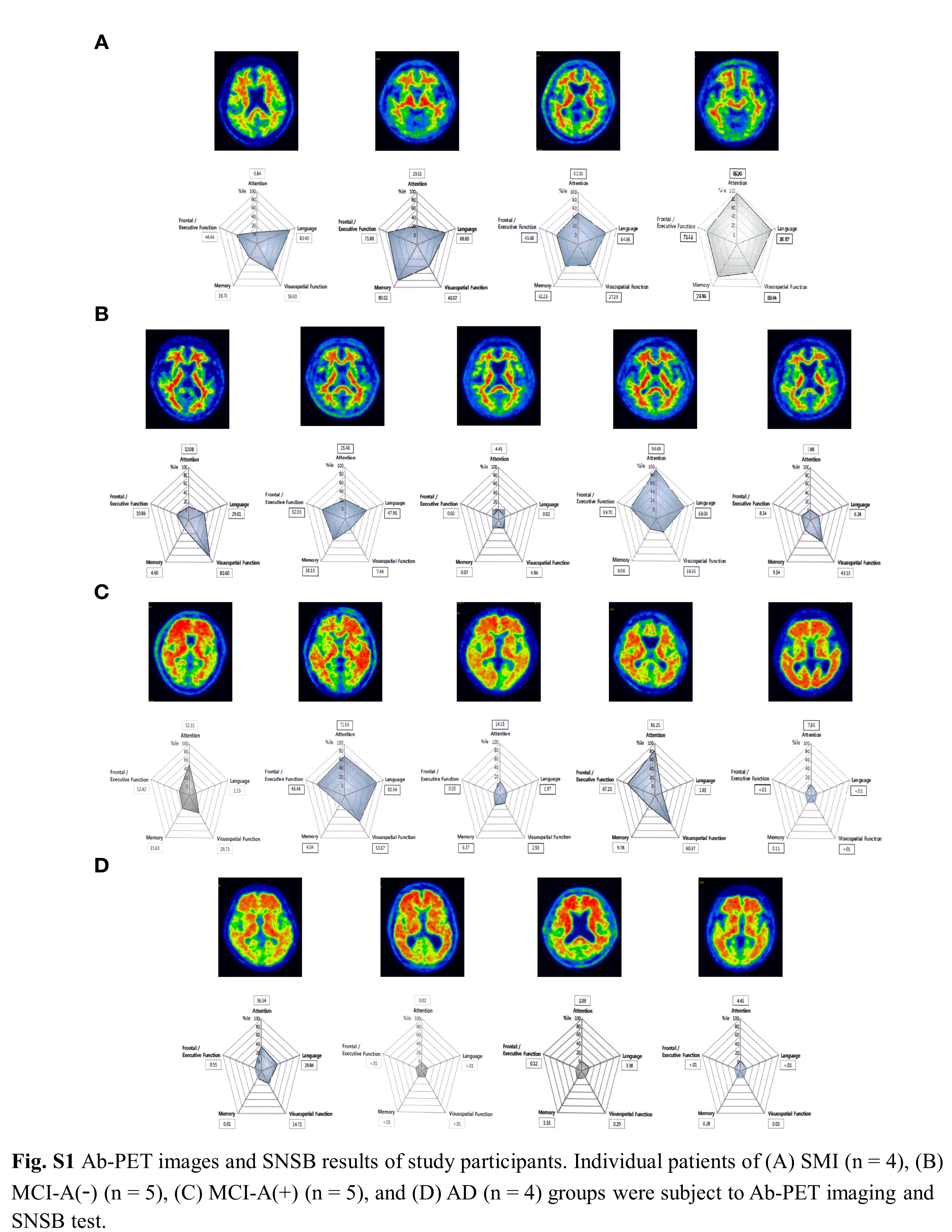

Supplement: Supplementary file 1 — Supplementary Material 1 Aβ-PET images and SNSB results of study participants [file 13041_2026_1294_MOESM1_ESM.tif]

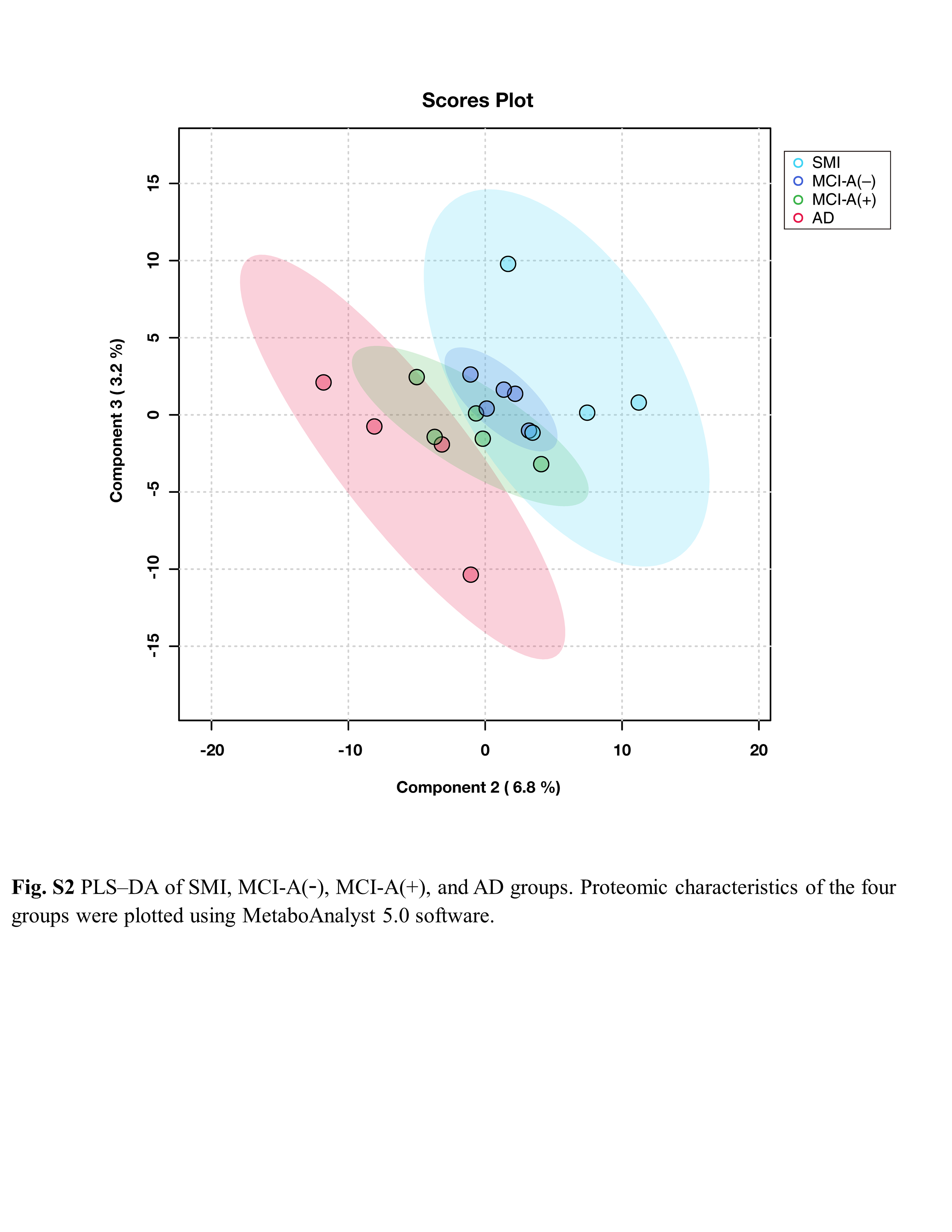

Supplement: Supplementary file 2 — Supplementary Material 2 PLS–DA of proteomic profiles from SMI, MCI-A(−), MCI-A(+), and AD groups [file 13041_2026_1294_MOESM2_ESM.tif]

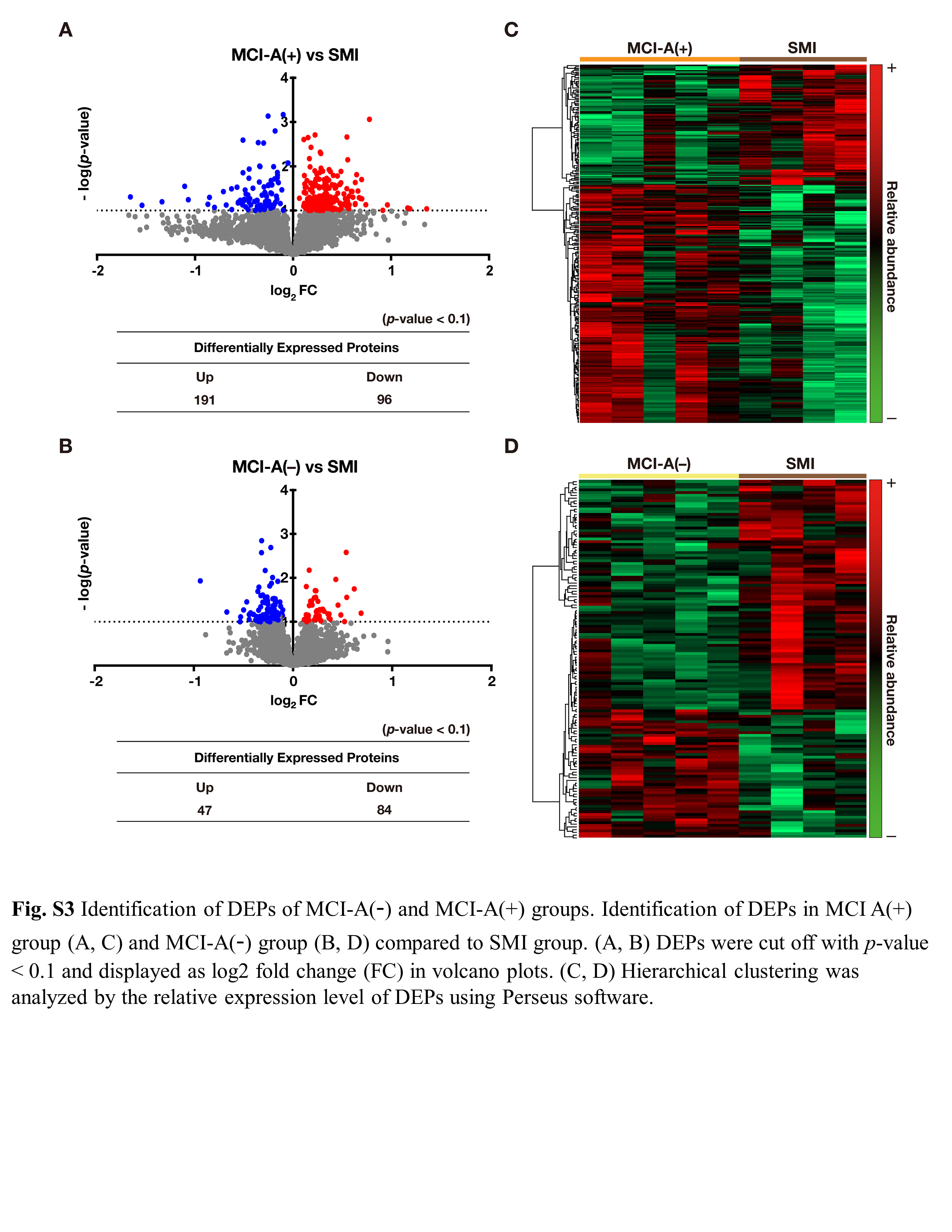

Supplement: Supplementary file 3 — Supplementary Material 3 Identification of DEPs in MCI subgroups compared with SMI [file 13041_2026_1294_MOESM3_ESM.tif]
